# SilenceREIN: seeking silencers on anchors of chromatin loops by deep graph neural networks

**DOI:** 10.1093/bib/bbad494

**Published:** 2024-01-02

**Authors:** Jian-Hua Pan, Pu-Feng Du

**Affiliations:** College of Intelligence and Computing, Tianjin University, Tianjin 300350, China; College of Intelligence and Computing, Tianjin University, Tianjin 300350, China

**Keywords:** silencer, regulatory element interaction network, graph neural network

## Abstract

Silencers are repressive *cis*-regulatory elements that play crucial roles in transcriptional regulation. Experimental methods for identifying silencers are always costly and time-consuming. Computational methods, which relies on genomic sequence features, have been introduced as alternative approaches. However, silencers do not have significant epigenomic signature. Therefore, we explore a new way to computationally identify silencers, by incorporating chromatin structural information. We propose the SilenceREIN method, which focuses on finding silencers on anchors of chromatin loops. By using graph neural networks, we extracted chromatin structural information from a regulatory element interaction network. SilenceREIN integrated the chromatin structural information with linear genomic signatures to find silencers. The predictive performance of SilenceREIN is comparable or better than other states-of-the-art methods. We performed a genome-wide scanning to systematically find silencers in human genome. Results suggest that silencers are widespread on anchors of chromatin loops. In addition, enrichment analysis of transcription factor binding motif support our prediction results. As far as we can tell, this is the first attempt to incorporate chromatin structural information in finding silencers. All datasets and source codes of SilenceREIN have been deposited in a GitHub repository (https://github.com/JianHPan/SilenceREIN).

## INTRODUCTION

The regulation of gene expression plays crucial roles in every living cell [1]. Biological processes, such as differentiation, proliferation and apoptosis, depend on proper spatial and temporal genes expressions. Abnormal gene expressions lead to various diseases [2, 3]. Gene expressions can be regulated at many levels. The transcription initiation, which is controlled by *cis*-regulatory elements and transcription factors (TFs), is the most basic regulation mechanism [4]. Identifying *cis*-regulatory elements, which is responsible for controlling transcription initiation, is of great importance to understand transcriptional regulations.

In eukaryotes, *cis*-regulatory elements include promoters, enhancers, silencers, insulators and many others. Promoters are located near and upstream of gene transcription start sites (TSSs). RNA polymerases bind to promoters to initiate gene transcriptions [5]. Enhancers and silencers locate upstream or downstream of TSS. The enhancer amplifies transcription by physically approaching its target promoter by a long-range chromatin loop [6]. Silencers may repress gene transcription also by approaching its target promoter by a long-range chromatin loop. The effects of silencers and enhancers create a dynamic balance to stabilize gene expressions.

The up-regulation and down-regulation of gene transcription are both crucial. However, the studies focused on silencers have lagged far behind those of enhancers. This is due to the limitation of available experimental technology for assaying and identifying silencers [7]. Recently, several experimental technologies, including massive parallel reporter assays (MPRAs) [8, 9], repressive ability measures of silencer elements (ReSE) [9] and self-transcribing active regulatory region sequencing (STARR-seq) [10], have been applied to find silencers in a high-throughput manner. A comprehensive database, which is called SilencerDB [11], has been released to deposit silencer records.

Despite that these assays are effective and efficient for finding silencers, they are still costly and time-consuming. Therefore, computational methods have been introduced as alternative approaches. Several studies have tried to use linear genomic or epigenomic features to find silencers. Huang *et al.* [12] identified silencers using correlations between DHS-H3K27me3 overlapping peaks and gene expression levels. Jayavelu *et al.* [8] utilized the sequence information of DNAs to train a gkmSVM [13] predictor. Zeng *et al.* [11] developed DeepSilencer for supplying data in the SilencerDB database. Recently, Huang *et al.* [14] proposed a multi-class convolutional neural network (CNN) model that predicts enhancers, silencers and regulatory neutral sequences.

All existing methods focus on using linear genomic or epigenomic features to find silencers. However, silencers often interact with other *cis*-regulatory elements to repress gene expressions. Long-range transcriptional regulatory elements, including enhancers, silencers, insulators and many others, may function through a DNA-looping mechanism that brings regulatory elements into proximity [4]. It can be inferred that these regulatory elements are frequently found at the anchors of chromatin loops. Silencers can be classified into proximal and distal silencers depending on their position relative to the gene promoter [15]. Promoter-proximal silencers are usually position-dependent and contain binding sites for repressor proteins that can repress gene expression by inhibiting transcriptional machinery binding or function [16]. Promoter-distal silencers are orientation- and position-independent, interacting with target promoters through chromatin loops to repress gene expression [4, 16]. For example, a silencer, which locates in the upstream of the PAI-2 (plasminogen activator inhibitor type-2) gene promoter, was found to repress the gene expression by binding the promoter. Another positive *cis*-regulatory element, which is located in the upstream of that silencer, appears to overcome the repression by binding that silencer [17]. In addition, Pang *et al.* [9] integrated the silencers identified from K562 cell with Hi-C data from human primary blood cells. They found that silencers interact physically with approximately 4000 promoter regions. Silencers may also interact with enhancer. For example, there is a silencer downstream the CD4 gene and an enhancer upstream. When the gene is repressed, the enhancer binds to the silencer. When the gene expresses, the enhancer binds to the promoter [18]. These regulation effects can only be achieved with special chromatin structures. Recently, Cai *et al.* [19] identified MRRs (H3K27me3-rich regions) with exquisite enrichment of H3K27me3 as putative human silencers. They also found that MRRs were highly associated with chromatin interactions. MRRs showed extensive looping within clusters and to distant genes. Genes located proximal to MRRs and genes distal to MRRs were both associated with low gene expression to a similar extent, indicating that silencers can function effectively across long distances through chromatin looping. These reports implied that chromatin interactions can be very useful information in finding silencers.

Long-range interactions between specific pairs of anchors can be evaluated with Chromosome Conformation Capture (3C) [20], which uses proximity ligation of cross-linked genomic loci *in vivo* to estimate contact frequencies [21]. The Hi-C protocol coupled proximity-based ligation with massively parallel sequencing [22]. The ChIA-PET [23] and HiChIP [24] technologies can detect loops bound to target proteins through chromatin immunoprecipitation steps. These experimental technologies enabled us to integrate chromatin interaction data in finding silencers.

In this work, we assumed that silencers, as well as other *cis*-regulatory elements, tends to locate at the anchors of chromatin loops. Dynamic chromatin interactions provide useful information to find silencers. We constructed a regulatory element interaction network by integrating chromatin structure data and regulatory elements data. We extracted the regulatory element interaction network structure features using a graph neural network model. Various linear genomic and epigenomic features are also integrated with a deep convolutional neural network model. We name our model as SilenceREIN (Silencers on the Regulatory Element Interaction Network). SilenceREIN is the first computational model to find silencers using regulatory element interactions. SilenceREIN has a comparable or better predictive performance than all state-of-the-art methods. A genome-wide scanning of silencers was performed, indicating that regulatory elements are commonly found at anchors of chromatin loops.

## MATERIALS AND METHODS

### Dataset curations

We focused on finding silencers in the K562 cell line. We collected 6924 silencers of K562 cell line from the SilencerDB [11]. Since supervised machine learning models were applied in our study, reliable negative samples are required. We collected 1695 enhancers in K562 cells from the NET-CAGE study [25], 29 598 human promoters from the EPD (Eukaryotic Promoter Database) database [26] and 2000 non-silencer sequences, which are identified by the STARR-seq assay, from Jayavelu *et al.* [8]. These non-silencer sequences were also applied in training DeepSilencer in the SilencerDB study [11]. We term all these sequences as negatives in our study. To construct the regulatory element interaction network, we obtained ChIA-PET-based chromatin conformation datasets from the ENCODE database. Both CTCF and POLR2A mediated chromatin interaction data were considered.

Histone modification signals were extracted from the reference epigenome ENCSR532GIR in the ENCODE database. The TF binding signals were extracted from various K562-based experiments in the ENCODE database. The histone modifications, including H3K9me3, H3K27me3, H3K27ac, H3K36me3, H3K4me3, H3K4me1, H3K79me2, H2AFZ, H4K20me1, H3K4me2, H3K9ac and H3K9me1 were considered in our study. The POLR2A, CTCF, ZNF143, SMC3 and RAD21 TF-binding signals were utilized in our study. All records were collected from K562 cells. We term this dataset as the primary benchmarking dataset.

To investigate how ChIP-seq data from diverse sources affect the performance of SlienceREIN, we curated another dataset from the ENCODE database, in which all records are produced by the Avocado pipeline [27] from a unique source. Due to the limitation of available data, the H3K9me1 and ZNF143 binding data were missing in this dataset. We use zeros to fill the gaps when generating features. We term this dataset as the alternative benchmarking dataset in our work. Raw data sources are recorded in Table 1.

**Table 1:** The dataset sources

Category	Type	Primary^a^	Alternative^b^
Histone modification ChIP-seq	H3K9me3	ENCFF632NQA	ENCFF238HRU
	H3K27me3	ENCFF582IMB	ENCFF073AOF
	H3K27ac	ENCFF465GBD	ENCFF180ADZ
	H3K36me3	ENCFF605EVL	ENCFF173IRF
	H3K4me3	ENCFF767UON	ENCFF549UCT
	H3K4me1	ENCFF457URZ	ENCFF756PXB
	H3K79me2	ENCFF334HSS	ENCFF049GYN
	H2AFZ	ENCFF202EVH	ENCFF908NCE
	H4K20me1	ENCFF694ODT	ENCFF589UOY
	H3K4me2	ENCFF054RSU	ENCFF205XIA
	H3K9ac	ENCFF239EBH	ENCFF905EHT
	H3K9me1	ENCFF220RGS	N.A.
TF ChIP-seq	RAD21	ENCFF377CPT	ENCFF803BJP
	POLR2A	ENCFF585IAR	ENCFF468JNP
	SMC3	ENCFF596CNE	ENCFF940GLX
	ZNF143	ENCFF886EZS	N.A.
	CTCF	ENCFF433VSV	ENCFF971EFJ
*Cis-*regulatory elements	Silencer	SilencerDB (6924)	SilencerDB (6924)
	Promoter	EPD (29 598)	EPD (29 598)
	Enhancer	NET-CAGE (1695)	NET-CAGE (1695)
	Non-silencer	Jayavelu *et al.* [8](2000)	Jayavelu *et al.* [8](2000)
ChIA-PET	CTCF	ENCFF118PBQ	ENCFF118PBQ
		ENCFF607PZX	ENCFF607PZX
	POLR2A	ENCFF511QFN	ENCFF511QFN
		ENCFF759YBZ	ENCFF759YBZ
		ENCFF030PMM	ENCFF030PMM

^a^The primary benchmarking datasets.

^b^The alternative benchmarking datasets. N.A. in this column indicates that type of data is missing. The numbers after names of database are records in this study.

Quality controls were performed in curating both datasets. All *cis*-regulatory elements were mapped to the hg38 reference genome using standard liftover utility. ChIP-seq series in both datasets were mapped to the hg38 reference genome. They are processed with standard ENCODE pipeline, which produces replicable and comparable results [28]. ChIA-PET datasets were also prepared by the ENCODE standard pipeline ChIA-PIPE, which automated quality control assessment for each dataset.

### Overview of the SilenceREIN protocol

**Figure 1:**
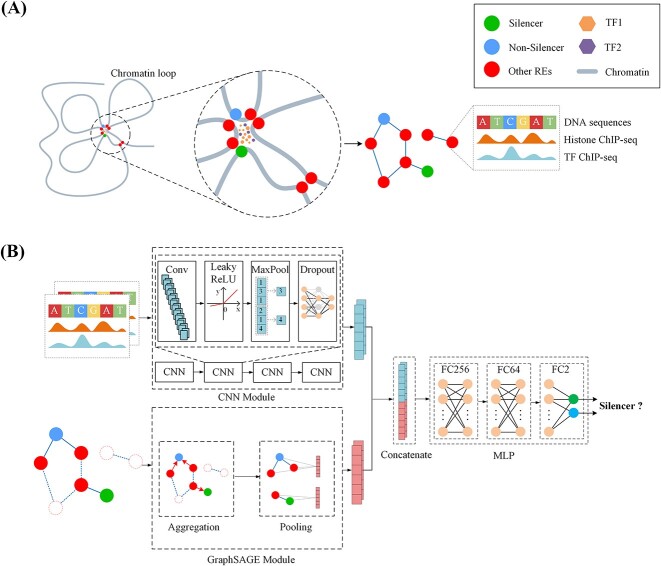
The overview of the SilenceREIN method. (**A**) The construction procedures of the regulatory element interaction network. Anchors of chromatin loops are usually preferred by transcription factors. These anchors are represented as nodes in a network. The regulatory elements are marked as node attributes. (**B**) Regulatory element interaction network structure information is captured by the GraphSAGE module. Linear genomic signatures are refined by a CNN module. The features from the CNN module and the GraphSAGE module are concatenated. An MLP classifier is trained to identify silencers.

The workflow of SilenceREIN is illustrated in Figure 1. The SilenceREIN method utilizes two types of information, the structure of the regulatory element interaction network and the linear genomic features of regulatory element sequences. The structural features of the regulatory element interaction network are extracted by a GraphSAGE module, which is composed by an aggregation layer and a pooling layer. The linear genomic signatures, which contain DNA sequence information, histone modification and TF binding signals, are processed by a CNN module. The CNN module contains four consecutive units. Each unit is composed by a convolutional layer, a LeakyReLU layer, a max pooling layer and a dropout layer. The output of the CNN module and the output of the GraphSAGE module are concatenated together to serve as the input of an Multilayer Perceptron (MLP) classifier. The MLP classifier contains three fully connected layers, with 256, 64 and 2 neurons, respectively.

### Regulatory element interaction network construction

**Figure 2:**
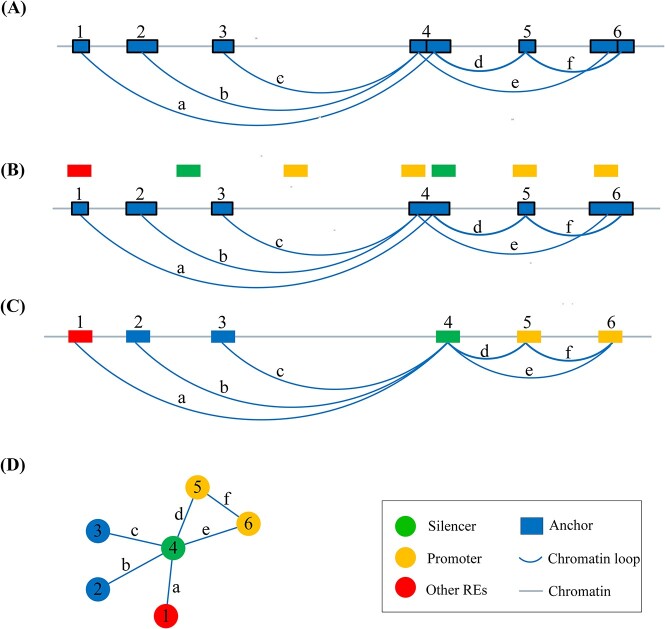
Workflow for constructing the regulatory element interaction network. (**A**) Sort all chromatin loop anchors. Cluster overlapping anchors and assign each cluster a unique cluster ID, with anchors within the same cluster sharing the same ID. Cluster IDs are indicated with numbers above the anchors (1 ~ 6). Clusters 4 and 6 consist of two anchors each, while the rest consist of one anchor. (**B**) Sequentially merge overlapping anchors according to their cluster IDs. Normalize the length of *cis*-regulatory elements to 600 nt. Overlaps are made between the normalized *cis*-regulatory elements and the anchors, considering the elements to be located on the anchors when the overlap exceeds half of an element’s length. (**C**) Label each anchor type based on the priority of silencer, non-silencer, enhancer and promoter. (**D**) In the regulatory element interaction network, use the cluster ID as the node ID and establish edges between node pairs based on chromatin loops (a ~ f). Construct isolated subgraph components using the BFS algorithm.

We construct high-resolution regulatory element interaction network using ChIA-PET based chromatin interaction data. Figure 2 illustrates the workflow for constructing regulatory element interaction network. Chromatin loops from different datasets were pooled. First, we utilized ChiaSigScaled, which is an optimal implementation of ChiaSig [29], to identify statistically significant ChIA-PET interactions. We kept chromatin loops with two anchors that are both less than 1000 nt in length. We obtained 777 358 chromatin loops with 1 549 647 anchors under this condition. The median anchor length is roughly 640 nt.

Each anchor is represented as a node in the regulatory element interaction network. Each pair of anchors are connected in the network. We reduce the network by merging overlapping anchors. If two anchors are overlapped with each other in genome, they were merged into one single anchor, which replaces two overlapping anchors. Edges to two overlapping anchors are all connected to the joint one. Edges between two overlapping anchors created self-loops. This reduction procedure was performed globally and iteratively until no anchor merging can be performed. We finally constructed a network with 761 371 nodes and 1 526 246 edges. The median anchor length after this reduction is about 650 nt.

Regulatory elements are marked on nodes as an attribute. Every regulatory element was normalized to 600 nt by keeping 300-nt flanking sequences on both up- and down-stream the center point of the element. If a regulatory element has more than 300 nt overlapping with an anchor, we mark the corresponding node to have that regulatory element type attribute. If an anchor is overlapped with multiple types of regulatory elements, we mark the corresponding nodes in this priority: silencer, non-silencer, enhancer and promoter.

We explore this network with the BFS (breath-first-search) algorithm. We find that this network can be dissected to several isolated subgraph components. Components with more than 100 nodes were kept in our study. Others were discarded. Finally, we have 24 components containing 421 761 nodes and 1 118 754 edges. 2365 nodes were marked as silencers, 390 as non-silencers, 1019 as enhancers, 11 279 as promoters and 406 708 as unlabeled. All chromosomes except chromosome 13 contain one component each. Chromosome 13 contain two smaller components. The statistics of these components are listed in Table S1 in the supplementary materials. Since negatives are far more than silencers, we balanced the dataset by including all non-silencers, enhancers and 10% of promoters in each subgraph components as negative samples. This approach composes a balanced dataset to the comparable size as silencers. Eventually, we obtained a dataset containing 2365 positive and 2536 negative samples, respectively. Although we did not use the unlabeled nodes as negatives, some individual nodes may still have false labels. This is due the nature of silencers [30]. According to literatures [7, 8, 31–33], silencers may have different regulation roles in different contexts. Therefore, some negatives in our dataset may be silencers in other context and vice versa. However, our dataset represents the best effort we can make and the best data basis until today. We take our dataset as the ground truth for all downstream analysis.

### Regulatory element interaction network features

The structural feature of the regulatory element interaction network was extracted by the GraphSAGE module [34]. GraphSAGE is an inductive node embedding framework, which can efficiently generate feature embeddings from local network structures. GraphSAGE trains a set of aggregation functions that generate feature embeddings for every node by sampling and aggregating features from the enclosing subgraph of a node. We chose to use the average aggregation function as follows: 


(1) \begin{equation*} {\mathbf{h}}_v(k)=\sigma \left(\mathbf{W}m\left(\left\{{\mathbf{h}}_v\left(k-1\right)\right\}\cup \left\{{\mathbf{h}}_u\left(k-1\right)|\forall u\in N(v)\right\}\right)\right), \end{equation*}

where W is a trainable weight matrix, h*_v_*(*k*) is the representation of node *v* in the *k*-th sampling step, *N*(*v*) is the set of neighboring nodes of node *v*, *σ*(.) and activation function and *m*(.) is the aggregation function for calculating the center vector of a set. The final node representation h*_v_*(*k*) is used as the topology feature for node *v*.

### Regulatory element linear features

The linear genomic signatures contain two parts. The first part is the one-hot encoding of the DNA sequence, as follows: 


(2) \begin{equation*} \left\{\begin{array}{c}\mathbf{A}=\left[\begin{array}{cccc}1 & 0& 0& 0\end{array}\right]\\[5pt] {}\mathbf{C}=\left[\begin{array}{cccc}0& 0& 1& 0\end{array}\right]\\[5pt] {}\mathbf{G}=\left[\begin{array}{cccc}0& 0& 0& 1\end{array}\right]\\[5pt] {}\mathbf{T}=\left[\begin{array}{cccc}0& 1& 0& 0\end{array}\right]\\[5pt] {}\mathbf{N}=\left[\begin{array}{cccc}0& 0& 0& 0\end{array}\right]\end{array}\!\!\!\!\right., \end{equation*}

where A, T, G and C represent four types of standard nucleotide and N is the undetermined nucleotide. Since the length of the regulatory elements has been normalized to 600 nt, this part of feature is of 4 × 600 = 2400 dimension.

The second part contains histone modification and TF binding signals. We take 12 types of histone modifications and 5 types of TF bindings. This part of feature is of 17 × 600 = 10 200 dimension. These two parts are concatenated to form a (4 + 17) × 600 = 12 600-D feature vector as the regulatory element linear features.

### Davies–Bouldin index

We introduce the DBI (Davies–Bouldin Index, *d*) [35] to measure the extent of separation in the dataset, as follows: 


(3) \begin{equation*} d=\frac{v\left({T}_{+}\right)+v\left({T}_{-}\right)}{{\left|\mathbf{m}\left({T}_{+}\right)-\mathbf{m}\left({T}_{-}\right)\right|}^2}, \end{equation*}

where *T*_+_, *T*_−_ are the sets of positive and negative feature vectors, *v*(.) is the function to calculate variance of a set of vectors, m(.) is the function to calculate the center of a set of vectors and |.| is the operator to calculate the Euclidean distance between two vectors.

The function m(.) and *v*(.) are defined as follows:


(4) \begin{equation*} \mathbf{m}(X)=\frac{1}{n}\sum \limits_{i=1}^n{\mathbf{x}}_i, \end{equation*}

and 


(5) \begin{equation*} v(X)=\frac{1}{n}\sum \limits_{i=1}^n{\left|{\mathbf{x}}_i-\mathbf{m}(X)\right|}^2, \end{equation*}

where *X* represents a set of *n* vectors, as *X* = {*x*_1_, *x*_2_, …, *x_n_*}. Intuitively, a smaller DBI value indicates a better separation.

### Motif enrichment score

When we analyzed motifs on silencers that are predicted by SilenceREIN, we define a motif enrichment score as follows: 


(6) \begin{equation*} {e}_k=-\ln \left({c}_k/\sum \limits_{j=1}^m{c}_j\right), \end{equation*}

where *e_k_* is the motif enrichment score of the *k*th motif, *c_k_* is the match number of the *k*-th motif in a set of silencers and *m* is the total types of motifs that are matched.

### Aggregate peak analysis

We apply aggregate peak analysis (APA) [36, 37] to assess the enrichment of contacts between predicted silencers and promoters. Silencers, which are predicted by the negative binomial model, the correlation-based method, the SVM and the CNN were obtained from literatures [9, 12, 14, 38]. We obtained prediction results of gkmSVM [8] and DeepSilencer [11] from SilencerDB [11]. We mapped them to the hg38 reference genome using standard liftover utility. The promoters was download form EPD [26]. We downloaded CTCF ChIA-PET and POLR2A ChIA-PET data (ENCFF162SWZ, ENCFF448TGS) from ENCODE. Two-dimensional contact matrices were generated using Juicer tools [37]. Regulatory element interaction network was constructed as mentioned. Interactions between promoters and silencer predictions were obtained in the network. Statistics on the number of interactions between promoters and silencer predictions are recorded in Table S2 in supplementary materials. APA was performed. Results with resolutions of 5000, 10 000 and 25 000 bp were recorded in Table S3 in supplementary materials.

### HiChIP data processing

We explored the possibility of using HiChIP data for generating chromatin interactions. We generated a contact matrix and chromatin loops from H3K27ac HiChIP data, which are provided by Mumbach *et al.* [39]. HiChIP paired-end reads were aligned to the hg38 reference genome using the HiC-Pro pipeline [40]. Default settings were applied throughout the HiC-Pro pipeline. Reads were converted into a contact matrix using the hicpro2juicebox function after HiC-Pro filtering. We retrieve statistically significant interaction from HiChIP data using hichipper [41]. ChIP-seq peaks was provided by H3K27ac data (ENCFF544LXB).

### Performance measures

We applied Accuracy (Acc), Sensitivity (Sen), Specificity (Spe), Positive predictive value (PPV), F1-Score (*F*_1_) and Matthews Correlation Coefficient (MCC) to measure the performance of SilenceREIN. The statistics are defined as follows:


(7) \begin{equation*} Acc=\frac{TP+ TN}{TP+ TN+ FP+ FN}, \end{equation*}

(8) \begin{equation*} Sen=\frac{TP}{TP+ FN}, \end{equation*}

(9) \begin{equation*} Spe=\frac{TN}{TN+ FP}, \end{equation*}

(10) \begin{equation*} PPV=\frac{TP}{TP+ FP}, \end{equation*}

(11) \begin{equation*} {F}_1=\frac{2 PPVSen}{PPV+ Sen}, \end{equation*}

and


(12) \begin{equation*} MCC=\frac{TPTN- FNFP}{\sqrt{\left( TP+ FP\right)\left( TP+ FN\right)\left( TN+ FP\right)\left( TN+ FN\right)}}, \end{equation*}

where *TP*, *TN*, *FP* and *FN* are the number of true positives, true negatives, false positives and false negatives in the cross-validations, respectively. In addition, we applied the area under receiver operation characteristic curve (AUROC) and area under precision-recall curve (AUPR) to describe the performances of SilenceREIN. It is worth noting that sensitivity can be termed as recall, while PPV can be termed as precision.

### Parameter calibrations and system implementations

SilenceREIN was implemented using python, with PyTorch and PyTorh-geometric. The PyTorch-geometric was used to implement the GraphSAGE module.

We optimized the architectural configuration of the CNN and the GraphSAGE modules by manual trials. We tried CNN modules with three, four and five layers and various channel configurations for both the CNN and the GraphSAGE modules. In the optimization procedures, AUROC in cross-validation was used as the performance measure. We use the optimal configuration of the CNN and the GraphSAGE modules. We set the *in_channels* and *out_channels* of the first CNN unit to 21 and 100, respectively. For the following three CNN units, both their *in_channels* and *out_channels* were set to 100. In the GraphSAGE module, we set the *in_channels* and *out_channels* to 10 and 100, respectively. In the MLP classifier, the number of neuron nodes for three fully connected layers is 256, 64 and 2, respectively. A dropout rate of 0.2 was applied as default throughout all neural network modules.

Other parameters of SilenceREIN are epoch, learning rate decay strategy, initial learning rate (*l*), L2 regularization coefficient (*λ*) and class weight (*w*). The initial learning rate, L2 regularization coefficient and class weight were optimized using a grid search strategy. In the optimization procedures, AUROC in cross-validation was used as the performance measure. We scanned *l*∈{0.0001, 0.0003, 0.0005, 0.0007, 0.0009}, *λ*∈{0.001, 0.0001, 0.00001} and *w*∈{1.1, 1.2, 1.3}. The optimal values are *l* = 0.0001, *λ* = 0.0001 and *w* = 1.2. The learning rate decay strategy was the learning rate multiplied by a parameter *γ* = 0.95 whenever loss rises.

## RESULTS AND DISCUSSIONS

### Predictive performance evaluation and comparison

**Figure 3:**
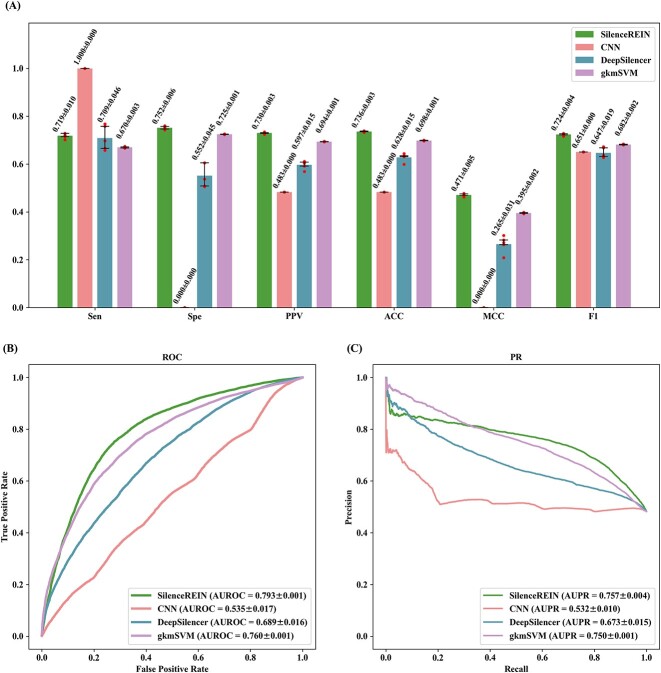
Performance comparisons between SilenceREIN and other state-of-the-art methods on the SilenceREIN dataset. (**A**) Performance comparison using 5-fold cross-validations. The standard deviations are estimated using five times 5-fold cross-validations with different random partitions. (**B**) The ROC curves of SilenceREIN and other state-of-the-art methods. (**C**) The PR curves of SilenceREIN and other state-of-the-art methods.

We evaluated predictive performances of SilenceREIN with comparisons to other state-of-the-art methods. All performance measures were estimated using 5-fold cross-validations on our primary benchmarking dataset. To mitigate the impact of random partitions in the cross-validation process, the 5-fold cross-validations were repeated five times for each method, with different random partitions each time. The average performance values of five-times 5-fold cross-validations were reported in Figure 3(A) with standard deviation values (SDs). SilenceREIN achieved better performances than DeepSilencer and gkmSVM in terms of most performance measures. The CNN method, which is provided in literature [14], gave a biased result. We believe that this is caused by some kind of over-fitting in the training process, as it is originally designed for a distinct category of silencers [14]. Although we have tried our best in adjusting parameters for that method, it does not work. To further evaluate the predictive performance comprehensively, we calculated the AUROC and AUPR for each method (Figure 3B and C). We performed *t*-tests to see whether SilenceREIN has a significant performance improvement over gkmSVM. SilenceREIN achieved an average AUROC of 0.793, with a 95% confidence interval of (0.791, 0.794). This is significantly higher than gkmSVM (*P* < 10^−9^, *t*-test, two-tails). The statistical significance values and confidence intervals for other comparison conditions and other performance measures are recorded in Tables S4 and S5.

Due to available resources, we compared SilenceREIN to works by Pang *et al.* [9] and Huang *et al.* [12] in a different way. APA was performed to assess the enrichment of silencer–promoter interactions on chromatin loop anchors. The results are presented in Table 2 and S2 and S3. Values, which are significantly above 1 for P2LL, and those, which are significantly above 0 for ZscoreLL, indicate enrichment. SilenceREIN achieved the highest P2LL and ZscoreLL in CTCF and POLR2A ChIA-PET data. This enrichment was not observed in H3K27ac HiChIP data, which indicates their frequent interaction with promoters and a negative correlation in chromatin loops with active gene expression.

**Table 2:** APA results

HiC	APA scores	NB model	Correlation-based model	SVM	SilenceREIN
CTCF ChIA-PET	P2LL^a^	4.45	N.A.	10.59	15.96
	ZscoreLL^b^	5.56	N.A.	14.12	147.11
POLR2A ChIA-PET	P2LL	2.31	N.A.	3.07	3.75
	ZscoreLL	7.92	N.A.	11.02	29.70
H3K27ac HiChIP	P2LL	0.57	N.A.	0.68	0.65
	ZscoreLL	−8.58	N.A.	−3.85	−9.13

^a^P2LL (Peak to Lower Left): the ratio of the central pixel to the mean of the mean of the pixels in the lower left corner.

^b^ZscoreLL (Zscore Lower Left): the Z-score of the of the central pixel relative to the all of the pixels in the lower-left corner.

It worth noting that silencers show a more significant enrichment in the CTCF ChIA-PET data. This implies a stronger association between silencers and the formation of CTCF-mediated chromatin loops. This observation is in consistent with earlier findings [8, 12, 42]. The enrichment in POLR2A ChIA-PET data may be associated with premature termination of transcription elongation [16, 43, 44].

For a comprehensive comparison, we also conducted APA for silencers that are predicted by gkmSVM [8], DeepSilencer [11] and CNN [14] (Table S3). SilenceREIN achieved the highest ZscoreLL in CTCF and POLR2A ChIA-PET data. Silencers, which are predicted by SilenceREIN, interact more frequently with promoters than silencers that are predicted by other methods, indicating they may exert more repressive function through chromatin loops.

### Performance analysis with different epigenomic signature datasets

**Figure 4:**
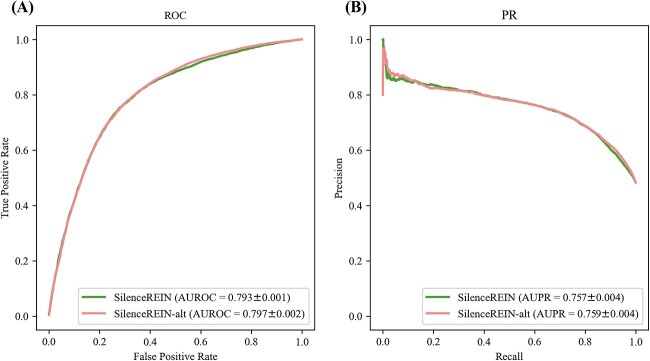
The impact of different ChIP-seq datasets. (**A**) The ROC curve of SilenceREIN on the primary and alternative dataset. (**B**) The PR curve of SilenceREIN on the primary and alternative dataset.

The histone modification and TF binding signals are based on ChIP-seq experiments. The ChIP-seq data processing pipeline may affect the performance of SilenceREIN. The primary benchmarking dataset contains ChIP-seq data from various sources. To investigate how ChIP-seq data from different sources affect SilenceREIN, we use the alternative benchmarking dataset, in which all ChIP-seq data are processed by the same protocol, as a contrast. The AUROC and AUPR values are calculated for SilenceREIN on both primary and alternative benchmarking datasets (Figure 4). Average values and SDs were computed from five times 5-fold cross-validation with different random partitions. SilenceREIN has a slightly higher performance on the alternative benchmarking dataset. However, considering the SDs, SilenceREIN shows no significant performance differences on the primary and alternative datasets (*P* > 0.05, two-tailed *t*-test). Therefore, we did not observe significant impact from different linear genomic signature data. This also implied that different ChIP-seq datasets do not change the intrinsic patterns that can be captured by machine learning algorithms.

SilenceREIN achieved a comparable performance on the alternative benchmarking dataset in the absence of some types of ChIP-seq profiles. Motivated by this observation, we explored how each type of ChIP-seq profile affect the predictive performance of SilenceREIN. We conducted 17 experiments, each involving only one type of ChIP-seq profile, while all remaining ChIP-seq profiles were replaced with zeros. As in Figure S1 in the supplementary materials, SilenceREIN works with an acceptable performance even with only one type of ChIP-seq profile. This suggests that SilenceREIN is a robust method for finding silencers with limited ChIP-seq data.

### Usefulness of the regulatory element interaction network features

SilenceREIN take regulatory element interaction network features into consideration. The predictive performance values in cross-validations suggest that silencers and other elements can be separated using sequence and network features. To quantitatively measure how good this separation is, we use the DBI with Euclidean distances [35]. On the primary dataset, SilenceREIN features have a DBI of about 2.36. When the network information was removed, the DBI increased to 3.27, which is about a 39% increment. On the alternative dataset, a DBI increment of 33% was also observed after removing network information. Therefore, on both primary and alternative datasets, SilenceREIN features are better clustered with chromatin structure information. The inclusion of the chromatin structure information increases the extent of separation.

**Figure 5:**
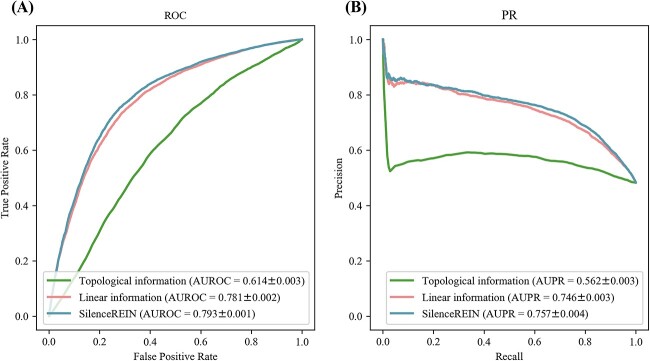
(**A**) ROC curves when only topology or linear information is used. (**B**) PR curves when only topology or linear information is used.

**Figure 6:**
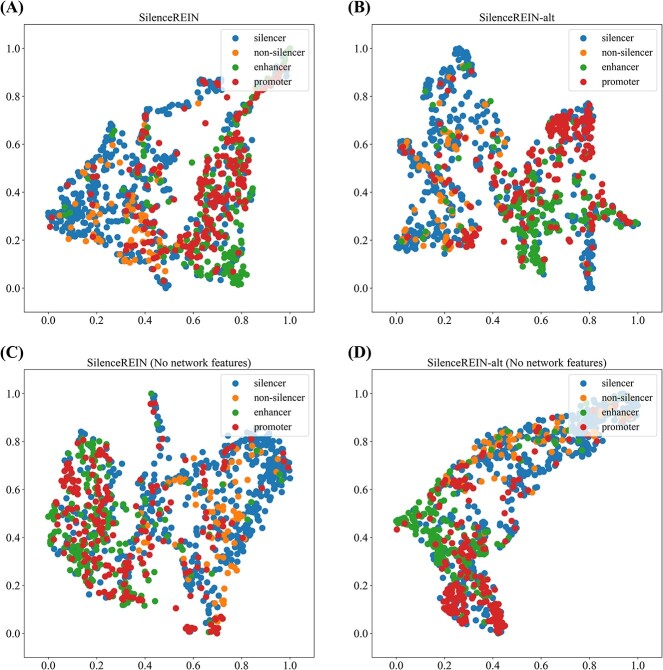
The UMAP visualization of feature vectors with and without the regulatory element interaction network information. For clarity, only 500 silencers and 500 negatives are randomly picked to present. On both primary and alternative datasets, network features improve the separation between silencers and negatives. (**A**) UMAP visualization of SilenceREIN feature vectors on the primary benchmarking dataset. (**B**) UMAP visualization of SilenceREIN feature vectors on the alternative benchmarking dataset. (**C**) UMAP visualization of SilenceREIN feature vectors on the primary benchmarking dataset without network features. (**D**) UMAP visualization of SilenceREIN feature vectors on the alternative benchmarking dataset without network features.

To further investigate the usefulness of the network features, we carried out an ablation analysis. Combining network features and linear information improves the performance of silencer prediction in cross-validations (Figure 5). Although the increment seems not much, it is still significant (AUROC *P* < 10^−4^, two-tailed *t*-test, AUPR *P* < 10^−2^, two-tailed *t*-test). In addition, we applied UMAP [45] to generate 2D scatter plots of silencers and negatives just before their features are fed into the MLP classifier. For clarity, 500 silencers and 500 negatives (Table S6 in supplementary materials) are randomly selected for generating the plots. Figure 6(A) and (B) presents features of silencers and negatives in the primary and alternative datasets, respectively. It is intuitive that most silencers and negatives are separately clustered. When the network information is removed, Figure 6(C) and (D) illustrate that both silencers and negatives spread to a wider range than Figure 6(A) and (B), creating more overlapping regions. Based on all above observations, we believe that the network features are useful in separating silencers and negatives.

### Genome-wide prediction of silencers on the anchors of chromatin loops

SilenceREIN focused on silencers with special chromatin structure features. We performed a genome-wide prediction to see how many silencers can be found with the chromatin structure condition. We train SilenceREIN with our primary benchmarking dataset. All nodes in the regulatory element interaction network without regulatory element attribute were used as a testing dataset. SilenceREIN reported 331 958 potential silencers among 406 708 nodes, which covers 81.62% of all nodes in the regulatory element interaction network. This result seems to be a severely over-estimated number, which raises the concern of over-fitted model in SilenceREIN. To eliminate this concern, we use gkmSVM to perform the same training and testing procedures. gkmSVM reported 267 522 potential silencers among the same 406 708 nodes. In other words, gkmSVM recognizes nearly 70% of nodes to be silencers. This number is also weirdly high. In addition, both methods share 233 876 predictions in common, which is nearly 58% of all nodes. We do not believe that SilenceREIN and gkmSVM are both over-fitted on our primary benchmarking dataset.

With a closer look, we can see that both SilenceREIN and gkmSVM are based on supervised machine-learning algorithms. SilenceREIN utilized linear epigenomic features, while gkmSVM used sequence statistics. However, as stated in various literatures [7, 30, 46], silencers have no common epigenomic signature, which makes them difficult to find. Different assays may find different sets of silencers, with little or no overlap [14]. In addition, existing studies have proposed that a large number of silencers may exist in human genome [8, 14]. Therefore, in this kind of blind genome-wide scanning, SilenceREIN and gkmSVM may have successfully separate silencers from other types of regulatory elements. They may not report exactly the same results, as their models capture different features of the genomic sequences or epigenomic signatures.

To further support this hypothetical explanation, we downloaded K562 cCRE (candidate *cis*-regulatory element) data from the SCREEN [28], including the candidate promoters, candidate enhancers and CTCF-bound cCREs. A total of 139 740 candidate *cis*-regulatory element were downloaded. We map candidate *cis*-regulatory elements to all our testing data. Only 27 671 nodes in the regulatory element interaction network were mapped, occupying on 6.8% of all nodes. This observation supports our explanation to the genome-wide scanning results, and the speculation of a massive number of silencers in human genome, particularly on the anchors of chromatin loops.

Another interesting observation is that more silencers are captured exclusively by SilenceREIN in the genome-wide scanning. We performed an independent test using SilencerDB records. We excluded those records that have been included in the training set of SilenceREIN and gkmSVM. Among the remaining 4535 records, 31 silencers were captured exclusively by SilenceREIN, while only 12 exclusively by gkmSVM. Moreover, we conducted a full-blind test involving 17 silencers in K562 cells (Table S7). These silencers are manually collected from literatures with experimental evidences [8, 12, 19]. None of them is included in the SilencerDB or the training dataset. Therefore, their information is not leaked to any training procedure. SilenceREIN exclusively captured 3 of 17 silencers (chr1:178,501,647-178,502,091, chr11:2,020,842-2,022,515, chr20:53,535,247-53,535,546), while gkmSVM captured none of them exclusively. These silencers, which are exclusively captured by SilenceREIN, are supported by experimental evidences. For example, chr1:178,501,647-178,502,091 exhibits a negative correlation in gene expression with the upstream 160 000 bp region of the *RASAL2* gene [12]. For another instance, 4C-seq confirmed that chr11:2,020,842-2,022,515 (MRR2-A1) have chromatin interactions with *IGF2*, which can function as a looping silencer to repress *IGF2* in human K562 cells [19]. In the original report of the gkmSVM method, these silencers were neither included nor captured by the gkmSVM method. Therefore, SilenceREIN provide a more comprehensive prediction for silencers that function through chromatin loops.

### Cross-annotation analysis with functional genome annotation models

ChromHMM [47] and Segway [48] are commonly applied for functional genome annotations. These tools are known as the SAGA (Semi-Automated Genome Annotation) tools. Their raw annotations need to be interpreted manually. Recently, Libbrecht *et al.* proposed a fully automated genome annotation tool [49]. It introduces machine learning–based models for interpreting genome annotations that are produced by SAGA tools. SAGA models and Libbrecht’s model were developed as common-purpose genome functional annotation tools. They are more capable in finding promoters and enhancers. Although repressive element labels, such as ‘ReprW’, ‘Repr1’ and ‘FacultativeHet’, can be assigned by these models, none of these models provide functional label ‘silencer’ directly. It is interesting to see how SilenceREIN predictions are correlated with annotations from these genome annotation models.

We collected ChromHMM, Segway and Libbrecht’s annotation labels and interpretations in K562 cells from literatures [49, 50]. All annotations were mapped to hg38 reference genome. Annotations from these models cover over 90% of the whole genome. It is natural that over 90% of SilenceREIN benchmarking dataset and over 90% of SilenceREIN predictions are annotated.

**Figure 7:**
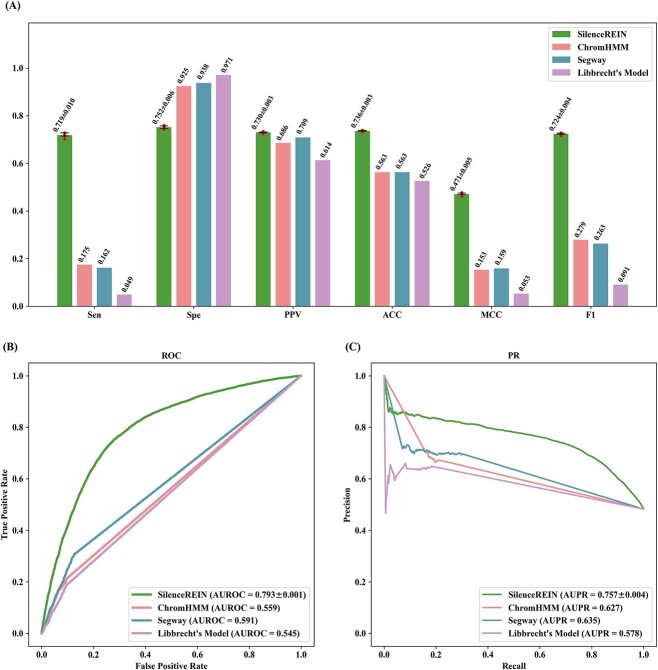
Performance comparisons between SilencerREIN and other genome annotation models. (**A**) Performance comparison of sensitivity, specificity, positive predictive value, Accuracy, MCC and F1-Score. (**B**) The ROC curves of SilenceREIN and other genome annotation models. (**C**) The PR curves of SilenceREIN and other genome annotation models.

We first validate if genome annotation models can correctly annotate the SilenceREIN benchmarking dataset. Silencers in the SilenceREIN benchmarking dataset are all with experimental evidence. This forms a ground truth to see the performances of genome annotations models. Since there is no explicit interpretation for which genome annotation is silencer, we took the ‘ReprW’, ‘Repr’ and ‘ReprD’ annotations by ChromHMM; the ‘Repr1’, ‘Repr2’, ‘Repr3’ and ‘Repr4’ annotations by Segway; and the ‘ConstitutiveHet’ and ‘FacultativeHet’ annotations by Libbrecht’s model, as the repressive element annotations. We calculate a repressive annotation score *r_s_* = *r* / *h* for each sample in the benchmarking dataset, where *r* is the region length overlapping with at least one repressive element annotation and *h* is the region length overlapping any annotation. If *r_s_* of a given sample is larger than a threshold *r_t_*, it is counted as a silencer and vice versa. Figure 7 presents the predictive performance values of ChromHMM, Segway and Libbrecht’s model with comparisons to SilenceREIN. Since genome annotation models do not produce results with randomness, no SD is recorded for ChromHMM, Segway or Libbrecht’s model in Figure 7. All point performance values (Sen, Spe, PPV, ACC, MCC and F1) are calculated with natural threshold *r_t_* = 0.5, while AUROC and AUPR with *r_t_* ∈ [0,1]. As a result, SilenceREIN has much better performances than all genome annotation models on the benchmarking dataset, especially in the term of AUROC.

**Figure 8:**
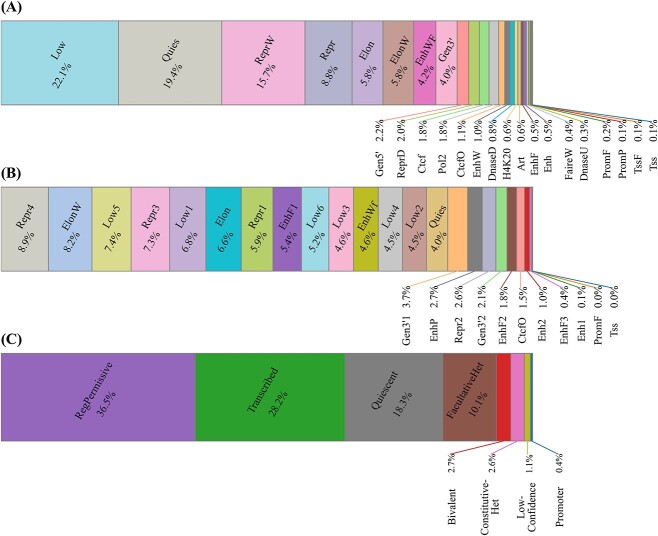
Cross-annotation analysis between SilenceREIN and genome annotation models. (**A**) The percentage of annotations from ChromHMM on SilenceREIN predictions. (**B**) The percentage of annotations from Segway on SilenceREIN predictions. (**C**) The percentage of annotations from Libbrecht’s model on SilenceREIN predictions.

We further measure the similarity genome-wide between SilenceREIN predictions and genome annotations from ChromHMM, Segway and Libbrecht’s model (Figure 8). ChromHMM annotations hit 26.5% of SilenceREIN predictions with the ‘ReprW’, ‘Repr’ or ‘ReprD’ label. This is significantly higher than expectations (*P* < 10^−5^, chi-square test), indicating that SilenceREIN predictions tend to correlate with the repressive element annotations by ChromHMM. Segway annotations hit 24.7% of SilenceREIN predictions with one of the four repressive element labels, including ‘Repr1’, ‘Repr2’, ‘Repr3’ and ‘Repr4’. This is again significantly higher than expectations (*P* < 10^−5^, chi-square test), indicating again that SilenceREIN predictions are in line with the functional genome annotations. Libbrecht’s annotations hit 12.7% of SilenceREIN predictions with the label ‘FacultativeHet’ or ‘ConstitutiveHet’, which is also significantly higher than expectations (*P* < 10^−5^, chi-square test). As noted by Libbrecht *et al.* [49], these regions are characterized by H3K27me3 or H3K9me3 signals. H3K27me3 and H3K9me3 had been associated with silencers by existing studies [7, 12, 19, 51].

**Figure 9:**
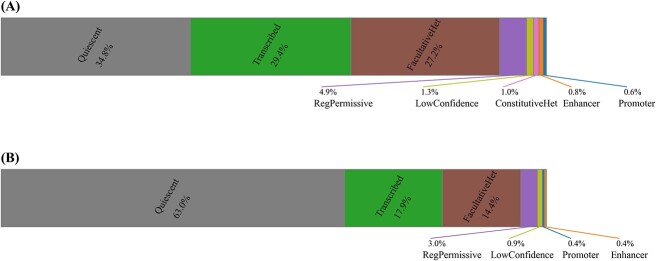
Cross-annotation analysis between SilenceREIN and Libbrecht’s model trained on SilenceREIN dataset. Pre-trained Libbrech’s model was applied to interpret ChromHMM and Segway raw annotation states that are produced using SilenceREIN dataset. (**A**) The percentage of annotations when interpreting ChromHMM raw annotation states. (**B**) The percentage of annotations when interpreting ChromHMM raw annotation states.

To further study the relationship between SilenceREIN predictions and Libbrecht’s annotations, we applied Libbrecht’s pre-trained model on our primary benchmarking dataset. Libbrecht’s pre-trained model was obtained from the pipeline of ENCSR019DPG in the ENCODE database. ChromHMM and Segway raw annotation states were generated solely using our primary benchmarking dataset. Libbrecht’s pre-trained model was applied to interpret these states. The results are presented in Figure 9. When Libbrecht’s model was applied in interpreting ChromHMM states, the repressive element labels hit 28.2% of SilenceREIN predictions, which is significantly higher than expectations (*P* < 10^−5^, chi-square test). When the Segway states were interpreted, they hit 14.4% of SilenceREIN predictions, which is again significantly higher than expectations (*P* < 10^−5^, chi-square test).

All above observations support that SilenceREIN produce silencer annotations that are in line with functional genome annotation models. Moreover, SilenceREIN can capture silencers that are missed by genome annotation models. We took SilenceREIN predictions without any of the repressive element annotation as silencers exclusive to SilenceREIN. We used the same 17 silencers with experimental evidences as in section Genome-wide prediction of silencers on the anchors of chromatin loops to perform full-blind evaluations to theses SilenceREIN exclusive predictions in the genome annotation context. SilenceREIN exclusively captured 2 out of these 17 silencers (chr5: 171,911,488-171,912,429, chr12: 65,983,501-65,984,072). 4C-seq confirmed that chr5: 171,911,488-171,912,429 (MRR1-A1) have chromatin interactions with *FGF18* and other genes, which can function as a looping silencer to repress *FGF18* as well as other genes in human K562 cells [19]. The region chr12:65,983,501-65,984,072 demonstrates a negative correlation in gene expression with the upstream 150 000 bp region of the *HMGA2* gene [12]. SilenceREIN is better at capturing silencers that function through chromatin loops.

Since genome annotation models are designed for common-purpose functional annotations, these results do not necessarily indicate a superior advantage of SilenceREIN. These results only provide evidence that SilenceREIN may capture some silencers that have been overlooked by genome annotation models, which makes SilenceREIN an indispensable supplementary to existing genome annotation models.

### Genome-wide TF-binding motif enrichment analysis on silencers

**Figure 10:**
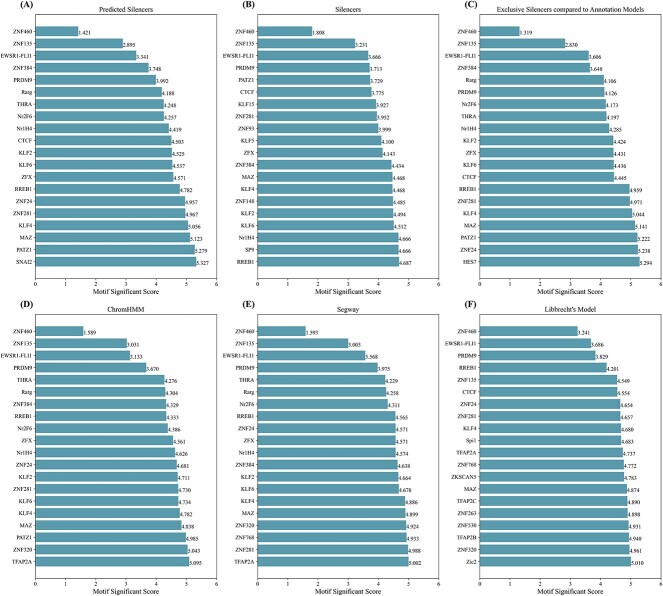
Motif enrichment analysis on the predicted and real silencers. In a genome-wide scanning, SilenceREIN predicted a number of silencers. The motifs on these silencers have a very similar distribution to those on the real silencers. For clarity, only the most frequent 20 motifs are presented in the figure. The number marked after each bar is the motif enrichment score, which is defined in Eq. (6). (**A**) The motif enrichment scores for the most frequent 20 motifs in the predicted silencers. (**B**) The motif enrichment scores for the most frequent 20 motifs in the real silencers. (**C**) The motif enrichment scores for the most frequent 20 motifs in the exclusive silencers compared to annotation models. (**D**) The motif enrichment scores for the most frequent 20 motifs in the repressive regions of ChromHMM’s annotations. (**E**) The motif enrichment scores for the most frequent 20 motifs in the repressive regions of Segway’s annotations. (**F**) The motif enrichment scores for the most frequent 20 motifs in the repressive regions of Libbrecht’s model annotations.

We further validated the genome-wide scanning results by motif enrichment analysis. TFs bind *cis*-regulatory elements to regulate gene expression. Activators bind enhancers to enhance gene expression. Repressors bind silencers to repress gene expressions [8]. It is interesting to investigate TF-binding motifs on silencers that are predicted by SilenceREIN. Since the total number of nodes in the regulatory element interaction network is too large, we randomly picked 10% of predicted silencers (Table S8 in supplementary materials) for this experiment. We performed motif analysis by scanning predicted silencers using FIMO [52], with motif data from JASPER [53]. Motif matches in the output with a *P*-value <10^−6^ were retrieved for further analysis. Figure 10(A) presents motif enrichment scores for the 20 most frequent transcription factor binding motifs in the analysis. As a contrast, we performed the same motif analysis on the silencers in our training dataset (Figure 10B). Figure 10(A) and (B) shares a list of most frequent TF-binding motifs, including ZNF460, ZNF135, EWSR1-FLI1, PRDM9, PATZ1, CTCF, ZNF281, ZFX, ZNF384, KLF4, KLF2, KLF6, Nr1H4 and RREB1. This result supports that the prediction of SilenceREIN has a similar preference of TF-binding motifs to the silencers in our training dataset. Particularly, PATZ1, CTCF and KLF4 have been associated with transcriptional repression functions [54–56].

In addition, ZNF460, ZNF135 and EWSR1-FLI1 are top three motifs in both Figure 10(A) and (B). PRDM9 ranks 5 and 4 in Figure 10(A) and (B), respectively. ZNF460 has been shown to be associated with transcriptional regulation involved in the development of cancers [57, 58]. EWSR1-FLI1 has been demonstrated to exhibit transcriptional silencer activity [59]. It is primarily associated with the repression of differentiation genes and further contributes to tumor development. PRDM9 is involved in the trimethylation of H3K4, a histone modification associated with gene activation and chromatin remodeling [60]. It has also been previously implicated in the regulation of chromatin structure and meiotic recombination [61, 62]. Its enrichment in silencers suggests that it may also contribute to the establishment and maintenance of repressive chromatin states at specific genomic loci. Although there is no direct evidence linking ZNF135 to transcriptional repression, we believe its enrichment in both predicted silencers and training silencers is not a coincidence, which may be worth further investigating in experimental genomics.

Moreover, we performed the same analysis on the repressive element label regions from all three genome annotation models in comparison, respectively, along with the silencer predictions exclusive to SilenceREIN. In Figure 10(C)–(F), all these models show similar motif enrichment patterns in their results, which is also similar to Figure 10(A) and (B) (e.g. ZNF460, ZNF135, EWSR1-FLI1, PRDM9). These results support that SilenceREIN exclusive predictions share similar motif preferences with other models.

### SilenceREIN on the HepG2 cell line

We performed all above analyses on the K562 cell line only. Limited by the data abundance [19], it is not feasible to consider various types of cell line. The only possible alternative cell line is the HepG2 cell. To our knowledge, massively parallel reporter experiments have been performed to detect silencers only in K562 cells and HepG2 cells so far. The number of silencers with experimental evidence in the HepG2 cell line is much less than that in the K562 cell line. We managed to extend SilenceREIN to work on the HepG2 cell line. The datasets used to construct regulatory element interaction network in HepG2 cells are recorded in Table S9 in supplementary materials. Using the same protocol in K562 cells, we successfully mapped 323 silencers, 0 non-silencers, 1603 enhancers, 9089 promoters and 155 062 unlabeled nodes in HepG2 cells. SilenceREIN achieved the best performance among all the methods in comparison (Figure S2 in supplementary materials). These results suggest that SilenceREIN has the potential to be extended to other cell lines.

### HiChIP-based regulatory element interaction network

Considering that H3K27ac HiChIP can identify functional regulatory elements [39], we constructed regulatory element interaction network based on H3K27ac HiChIP data. We construct regulatory element interaction network using the chromatin loops with FDR ≤ 0.01. Since lengths of the anchors are all greater than 1000 nt, we removed the restriction of keeping only the chromatin loops with anchor lengths less than 1000 nt when constructing the network. We mapped 575 silencers, 22 non-silencers, 732 enhancers, 5593 promoters and 10 416 unlabeled nodes. All non-silencer, enhancers and 10% of the promoters in each subgraph components were used as negative samples. With HiChIP data, SilenceREIN still achieved the best performance in comparisons to all state-of-the-art methods (Figure S3 in supplementary materials).

Although the predictive performances seem to be promising in comparisons, we still need to notice that the coverage of silencers is much less than using ChIA-PET data. This is due to the resolution of HiChIP experiments. Particularly, we also notice that the predictive performances drop when we use HiChIP to replace ChIA-PET data. These observations suggest that current HiChIP experiments do not have sufficient resolution to support SilenceREIN. However, we expect future development in the HiChIP experimental technology to improve its resolution, which may well support SilenceREIN. In the meantime, we also need to develop methods to work with low- resolution chromatin loops.

## CONCLUSION

Silencers repress the transcription of target genes. They have important functions in regulating gene expression. We developed SilenceREIN, which, to our knowledge, is the first computational method to incorporate chromatin structure information in recognizing silencers. Particularly, we focused on finding silencers on anchors of chromatin loops. By constructing a regulatory element interaction network, SilenceREIN successfully applied deep graph neural networks to extract chromatin structure information with regard to *cis*-regulatory element interactions. We trained and evaluated SilenceREIN on datasets from K562 cells and HepG2 cells. SilenceREIN achieved a comparable or better predictive performance than state-of-the-art methods. Genome-wide scanning and motif enrichment analysis supports that silencers may be commonly found on anchors of chromatin loops. SilenceREIN predictions are in line with existing genome annotation tools, with additional advantage of discovering silencers that function through chromatin loops. With sufficient data in future, SilenceREIN has the potential to be extended to other cell types.

**Key Points**

- A review on computational predictions of silencers in human genome
- The SilenceREIN method is the first attempt to integrate chromatin structure information in predicting silencers.
- According to a genome-wide scanning, a wide distribution of silencers was observed in the human genome.

## Supplementary Material

FigureS1_bbad494

FigureS2_bbad494

FigureS3_bbad494

silenceREIN_supp_TableS1_S9_1206_bbad494

## Data Availability

The code and data for reproducing the results of this paper is available in GitHub (https://github.com/JianHPan/SilenceREIN).

## Author Biographies

**Jian-Hua Pan** is a master student in the College of Intelligence and Computing, Tianjin University. His research interests focus on *cis*-regulatory element analysis.

**Pu-Feng Du** is an associate professor in the College of Intelligence and Computing, Tianjin University. His research interests include computational analysis of genomic and proteomic data.
