# Supplementary figures and images for: SilenceREIN: seeking silencers on anchors of chromatin loops by deep graph neural networks
Source: Brief Bioinform. 2024 Jan 2;25(1):bbad494. doi: 10.1093/bib/bbad494 (PMC10782921; doi:10.1093/bib/bbad494)

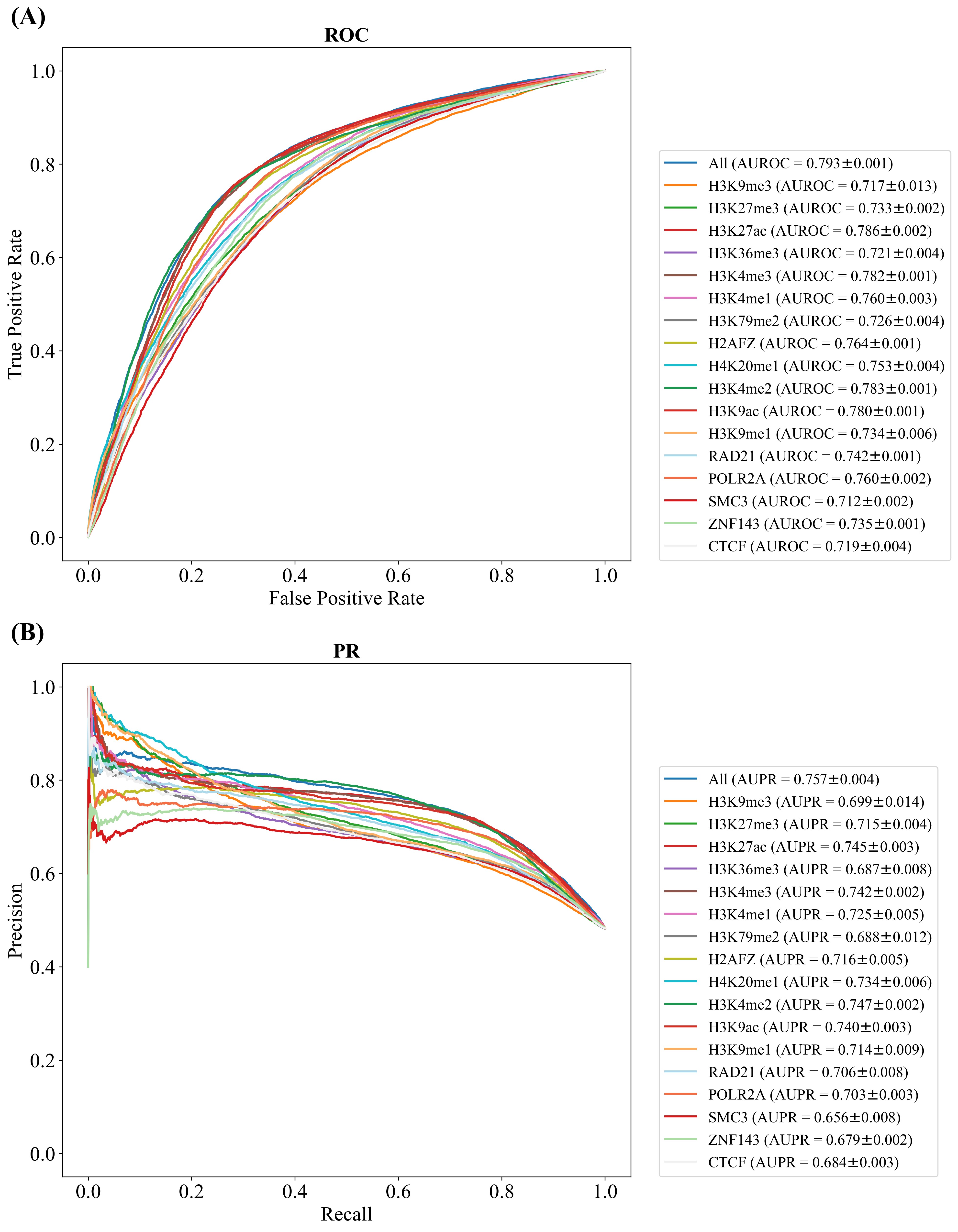

Supplement: FigureS1_bbad494 [file figures1_bbad494.jpeg]

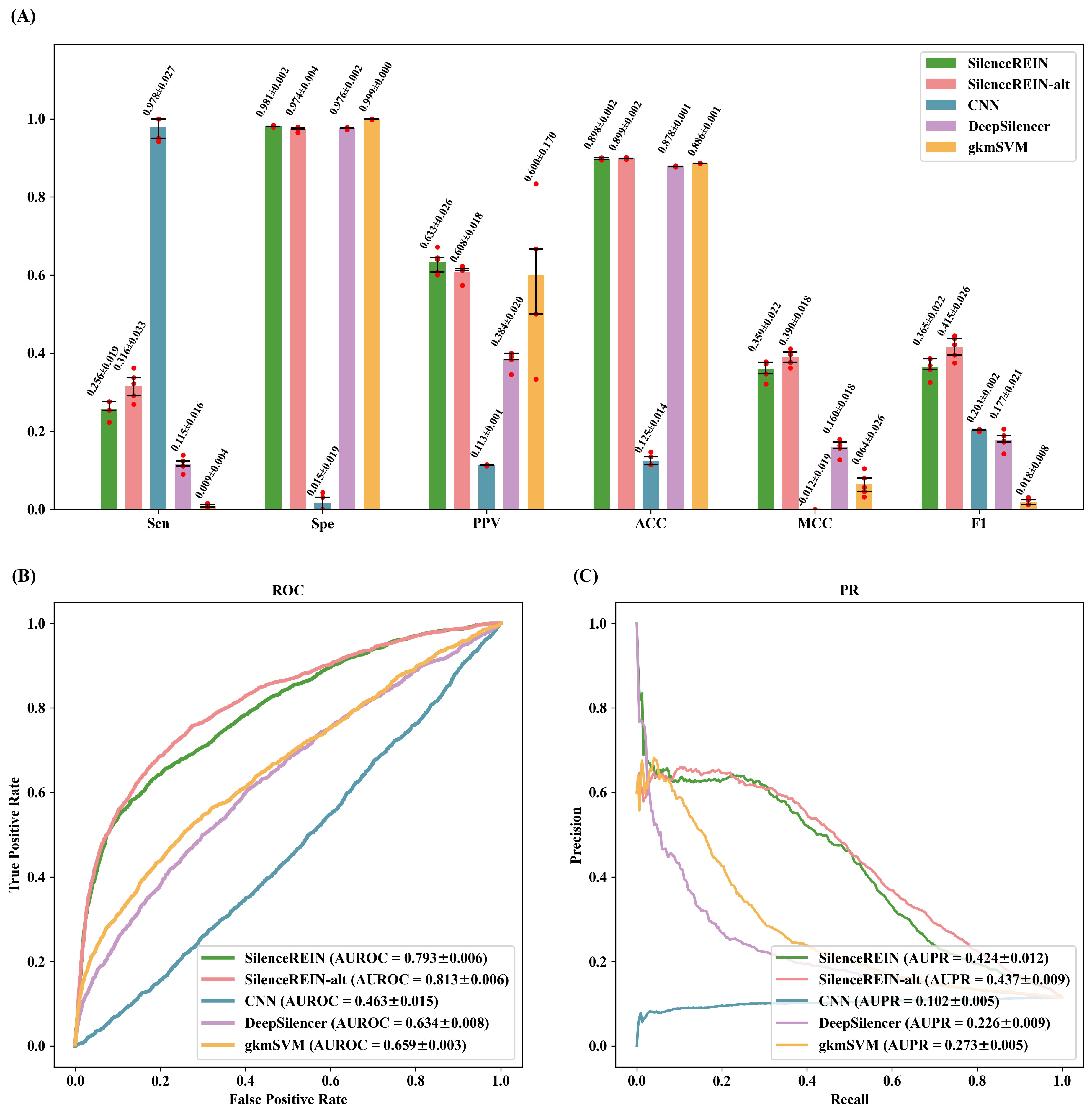

Supplement: FigureS2_bbad494 [file figures2_bbad494.jpeg]

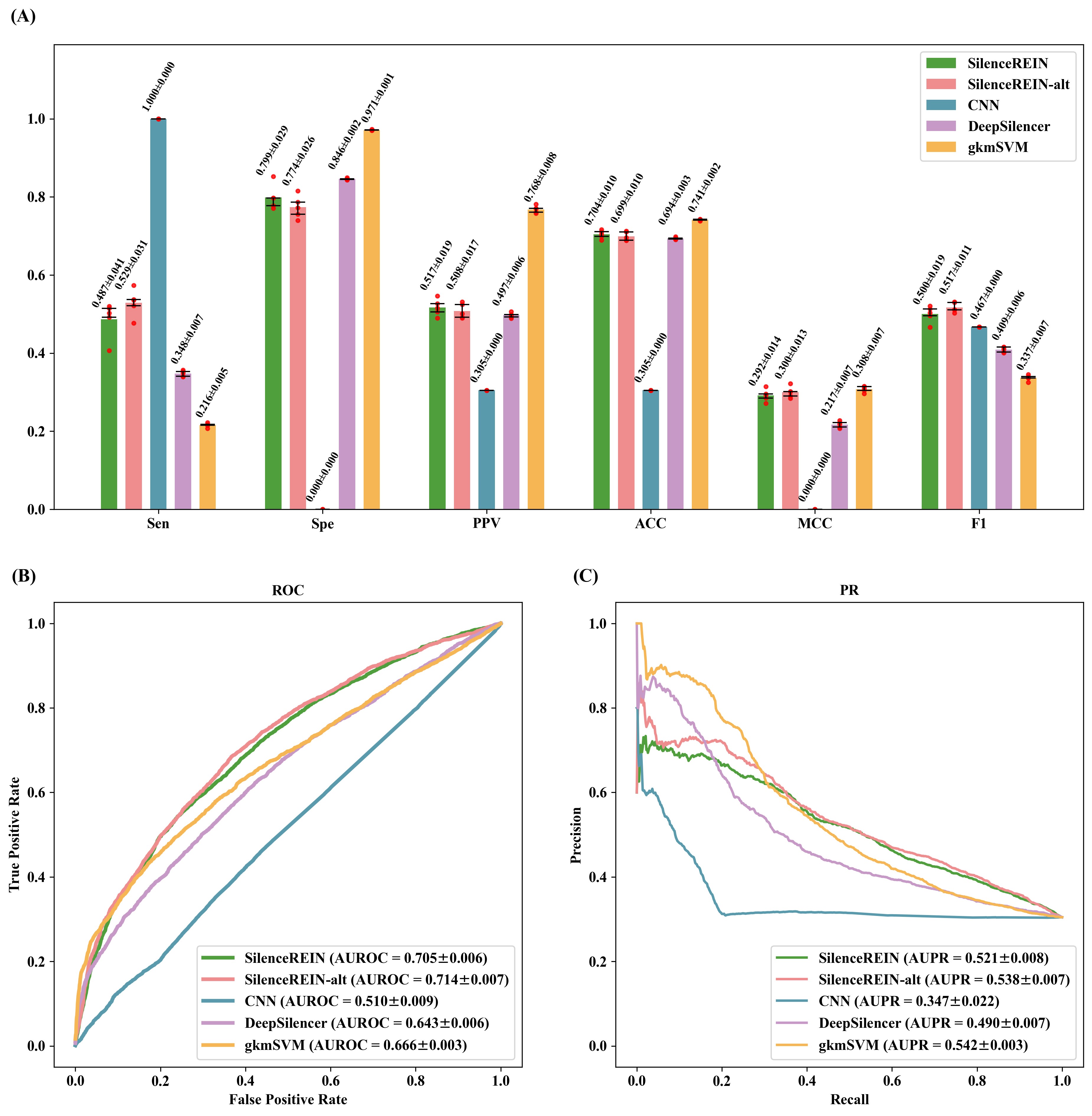

Supplement: FigureS3_bbad494 [file figures3_bbad494.jpeg]
